# The mediating effects of moral disengagement and aggressive humor style: Dark Triad traits and schadenfreude

**DOI:** 10.3389/fpsyg.2024.1361094

**Published:** 2024-07-01

**Authors:** Milad Sharafi Zadegan, Reza Pourhosein, Zohreh Azizi

**Affiliations:** ^1^Department of Psychology, University of Tehran, Tehran, Iran; ^2^Counseling Center, University of Tehran, Tehran, Iran

**Keywords:** aggressive humor style, Dark Triad, moral disengagement, schadenfreude, personality trait

## Abstract

**Background:**

While schadenfreude is commonly experienced in interpersonal relationships, it is generally considered immoral. Although previous research has explored the factors relating to schadenfreude, including certain personality traits, moral disengagement, and humor styles, our understanding of these factors remains limited. The present study examined the mediating effects of moral disengagement and aggressive humor style in the relationship between the Dark Triad traits and schadenfreude.

**Methods:**

In this study, a sample of 693 Iranian students (69.4% female), was recruited using a convenient sampling method, consisting of 330 undergraduate, 230 graduate, and 133 Ph.D. students. The trait schadenfreude scale, the short Dark Triad, the moral disengagement scale, and the aggressive humor style scale were measured through an online survey.

**Results:**

The study found that Machiavellianism, narcissism, and psychopathy were positively related to schadenfreude, aggressive humor style, and moral disengagement. Additionally, aggressive humor style and moral disengagement mediated the relationship between Machiavellianism, narcissism, and psychopathy with schadenfreude.

**Conclusion:**

This study provides insights into the complex relationships between Dark Triad traits, moral disengagement, aggressive humor style, and schadenfreude. The findings suggest that individuals with higher levels of Dark Triad traits may be more likely to experience pleasure from others’ misfortunes. Furthermore, the study highlights the importance of moral disengagement and aggressive humor style as potential mechanisms underlying the relationship between Dark Triad traits and schadenfreude. Further research should be done to explore the motivational factors influencing schadenfreude in specific settings, thereby elucidating these connections.

## 1 Introduction

Schadenfreude refers to the experience of joy at the misfortune of others (Greenier, 2021). While individuals may personally find schadenfreude enjoyable (Phillips et al., 2022), experiencing and expressing schadenfreude can result in immediate criticism from others (Gromet et al., 2016). Despite its moral unacceptability, schadenfreude is a common and normal human emotion (Schindler et al., 2015), reflecting the complex and multidimensional nature of human experience.

Schadenfreude has been actively studied over the past 30 years. Prior research has focused on identifying situations in which it is expressed, like social media (Watanabe, 2016; Cecconi et al., 2020), its correlation with other emotions from observing others’ misfortune, such as empathy (Ouwerkerk et al., 2018) and envy (Greenier, 2021), and the triggering factors of schadenfreude, including competition (Baren, 2017) and deservingness (Berndsen et al., 2017b). Although identifying these factors provides a foundation for understanding this common emotion in daily social relationships, this approach could not often capture the multifaceted nature of Schadenfreude, leading to recent research focusing on specific personality traits, such as Dark Triad (DT) traits, associated with schadenfreude (Erzi, 2020; Yee and Lee, 2022).

The DT traits (Paulhus and Williams, 2002) consists of narcissism, characterized by grandiosity, entitlement, and a lack of empathy; Machiavellianism, marked by manipulation and a focus on self-interest over moral considerations; and psychopathy, noted for impulsivity, callousness, and a lack of remorse. Many of these defining characteristics of the DT (e.g., lack of empathy or callousness, competitiveness, and hatred) are similar to behaviors that occur before experiencing schadenfreude. As a lack of empathy is common in Machiavellianism, narcissism, and psychopathy, this could provide a firm conceptual bond between DT traits and experiencing schadenfreude (Jonason et al., 2013). Besides, individuals with DT traits might prioritize their needs to satisfy their goals (Abell and Brewer, 2018) and consider other individuals failures as their benefit (Baren, 2017).

Moral disengagement is an underlying factor that might contribute to the relationship between DT traits and schadenfreude. In order to get rid of the external and internal outcomes of committing disruptive behaviors, individuals try to justify their disruptive and offensive behaviors through a psychological process called moral disengagement (Bandura et al., 1996). A few studies have shown a positive correlation between DT traits and moral disengagement (Egan et al., 2015; Erzi, 2020; Wu et al., 2020). Individuals who use moral disengagement mechanisms (e.g., ignoring or minimizing consequences) might experience less guilt or discomfort about their disruptive behaviors. This, in turn, could potentially influence their experience of schadenfreude. Although it does not seem that schadenfreude is similar to those disruptive behaviors, we can consider them as outcomes of DT traits (James et al., 2014; Porter et al., 2014) and immorality (Berndsen et al., 2017a). Even though there are inconsistencies in the relationship between Machiavellianism and moral disengagement in studies (Erzi, 2020; Lișman and Corneliu, 2023), it seems individuals with DT traits are likely more inclined to express schadenfreude through the moral disengagement mechanism.

As individuals with DT traits tend to undermine others (Goncalves and Campbell, 2014) so they consider others’ misery as a source of their entertainment (James et al., 2014); another underlying factor that could be introduced in the relationship between DT traits and schadenfreude is humor (Yee and Lee, 2022). Because it seems there is an overlap between humor and schadenfreude, we could posit schadenfreude as a humorous response (Gray, 2021). Previous studies concluded that schadenfreude results from social aggression (Erzi, 2020), which indicated schadenfreude is a reflection of internal tendencies, especially the tendency for social aversion (James et al., 2014). Thus it might be a response to stimulation by a specific humor style. This specific humor style is typical among individuals who feel insecure in social settings (Martin et al., 2003) and has a common feature that underlies schadenfreude. Reflecting on these findings, individuals who feel more insecure tend to express their poor ego through others’ failure (Brambilla and Riva, 2017). This phenomenon could be a social aggression expression that usually manifests as aggressive humor (Martin et al., 2003). Alternatively, studies have demonstrated that individuals with DT traits tend to use aggressive humor style to undermine others’ deeds (Martin et al., 2012). The above mentioned findings showed important bonds between DT traits and aggressive humor style with schadenfreude. Naturally, there have remained doubts and inconsistencies about narcissism and its relationship with aggressive humor style (Veselka et al., 2010; Yee and Lee, 2022).

Due to the observed findings in previous studies, a model suggested that examined mediating role of moral disengagement and aggressive humor style in the relationship between DT traits and schadenfreude. Although there is a substantial amount of studies on the mediating role of moral disengagement, only one study has examined the mediating role of humor style and used a small sample size (Yee and Lee, 2022). Therefore, considering a more substantial sample size, we tried to present results with more generalization capacity in this study. Besides, due to some inconsistencies in the relationship of DT traits with schadenfreude (James et al., 2014; Erzi, 2020), moral disengagement (Erzi, 2020; Lișman and Corneliu, 2023), and aggressive humor style (Veselka et al., 2010; Yee and Lee, 2022), it is expected that there is a positive relationship between Machiavellianism, narcissism, and psychopathy with schadenfreude and this relationship is mediated by moral disengagement and aggressive humor style.

The proposed model was constructed to test the following hypotheses.

*Hypothesis 1*: Dark Triad traits will be directly related to schadenfreude.

*Hypothesis 2*: Moral disengagement and aggressive humor style will be directly related to schadenfreude.

*Hypothesis 3*: Dark Triad traits will be directly related to moral disengagement and aggressive humor style.

*Hypothesis 4*: Moral disengagement and aggressive humor style mediate the relationship between Dark Triad traits and schadenfreude.

## 2 Materials and methods

### 2.1 Participants

The target participants in this study were Iranian students which due to the infeasibility of accessing all students, a convenient sampling method was employed (Haque, 2010). A total of 748 questionnaires was received through online distribution. Of these, 55 questionnaires were considered invalid and excluded because of the response time (2–15 min) or results (no obvious carelessness in completing the questionnaires); this constituted a validity rate of 92.65%. In the final sample of 693 participants, there were 330 undergraduate, 230 graduate, and 133 Ph.D. students. The female participants (*N* = 481, Mean = 24.85, SD = 4.13) and male participants (*N* = 212, Mean = 25.37, SD = 4.28) were aged between 18 and 37 years and 18–36 years, respectively.

### 2.2 Materials

#### 2.2.1 Trait schadenfreude scale

Schadenfreude was measured by Trait Schadenfreude Scale (Baren, 2017), a 24-item self-report questionnaire. The participants are asked to express their agreement to items from 1 (strongly disagree) to 7 (strongly agree). Barren (Baren, 2017) reported 0.92 for internal consistency and 0.90 for the test–retest reliability of this scale. Convergent validity with the dark personality scale was 0.51, with the envy scale 0.40, divergent validity with the self-esteem scale was −0.29, and empathy scale was −0.37. The Persian version of the Trait Schadenfreude Scale showed excellent internal consistency (α = 0.94) and construct validity (RMSEA = 0.070, CFI = 0.911, IFI = 0.912, χ^2^/df = 4.362) in the present study.

#### 2.2.2 Short Dark Triad

Dark Triad was measured by Short Dark Triad (Jones and Paulhus, 2014), including Machiavellianism, narcissism, and psychopathy. Each subscale has nine items, answered from 1 (strongly disagree) to 7 (strongly agree). Jones and Paulhus (2014) reported 0.73 to 0.78 for the internal consistency coefficient. In this study, the Persian version of this questionnaire was used, and its Cronbach’s alpha for Machiavellianism, narcissism, and psychopathy was 0.71, 0.82, and 0.69, respectively. The construct validity was also confirmed, with the following indices: RMSEA = 0.05, NFI = 0.98, RFI = 0.98, and χ^2^/df = 1.23 (Amiri and Yaghoobi, 2016).

#### 2.2.3 Moral disengagement scale

Moral disengagement was measured by the Moral disengagement scale (Moore et al., 2012). This scale consists of 8 items, and each item is scored from 1 (strongly disagree) to 7 (strongly agree), yielding a total score range of 8–56. Moore et al. (2012) reported the reliability of this scale using Cronbach’s alpha (α = 0.80) and examined its validity by confirmatory factor analysis. The fit indices showed that the scale had good construct validity (χ^2^/df = 1.35, SRMR = 0.04, NFI = 0.98, CFI = 0.99, RMSEA = 0.04). The Persian version of the scale also showed good internal consistency (α = 0.80) and validity (RMSEA = 0.07, CFI = 0.95, IFI = 0.95, X2/df = 4.47) in this study.

#### 2.2.4 Humor styles questionnaire

Aggressive humor style was measured by the 32-item humor styles questionnaire (Martin et al., 2003), which includes affiliative, self-enhancing, aggressive, and self-defeating. Each item is scored on a 7-point scale from extremely disagree (1) to extremely agree (7). Martin et al. (2003) reported the reliability of humor styles subscales 0.85, 0.81, 0.80, and 0.82, respectively. The Persian version of this questionnaire also demonstrated satisfactory reliability (Cronbach’s alpha between 0.70 and 0.73) and structural validity (RMSEA = 0.04, GFI = 0.90, CFI = 0.92, IFI = 0.90, X2/df = 1.55) (Sevari, 2018). In this study, only the aggressive humor style was used.

### 2.3 Procedure

Initially, an online version of the questionnaire was distributed to the participants through social media platforms, including Instagram, WhatsApp, and Telegram. The participants were provided with detailed instructions regarding the completion process, the voluntary nature of their participation, and the confidentiality of their responses. Informed consent was obtained from all participants before they proceeded to complete the questionnaires. As an incentive, the students received phone credit for their participation in the study. The study was approved by the Research Ethics Committee of the Faculty of Psychology and Education, Tehran University (Ethics ID: IR.UT.PSYEDU.REC.1401.043), and all research stages were conducted in accordance with the Ethics Standards of the Helsinki Declaration.

### 2.4 Analysis plan

Firstly, SPSS 22.0 was employed to assess data normality, identify potential outliers, generate descriptive statistics, and Pearson correlation coefficients among the study variables. A structural equation model with maximum likelihood and full information maximum likelihood approach was used in AMOS 20.0. Model fit was evaluated based on the Chi-square ratio (χ^2^/df), Comparative Fit Index (CFI), Incremental Fit Index (IFI), Tucker-Lewis Index (TLI), and Root Mean Square Error of Approximation (RMSEA). Acceptable model fit indices are characterized by χ^2^/df < 3, CFI > 0.90, IFI > 0.90, TLI > 0.90, and RMSEA <0.08 (Hu and Bentler, 1999). Additionally, the bootstrapping technique with a 95% confidence interval (CI) from 5,000 resamples was used to estimate direct and indirect effects.

## 3 Results

### 3.1 Preliminary analysis

To ensure data quality and establish preconditions, initial assessments were conducted. Notably, due to the structured nature of the online questionnaires, all participants were required to answer all questions before submitting their responses, resulting in no missing data. Skewness and kurtosis were employed to examine the normality assumption. The obtained values for skewness (−1.09 to 1.26) and kurtosis (−0.60 to 1.90) fell within the recommended ranges of ±3 for skewness and ±5 for kurtosis (Hair et al., 2006), indicating the normality of the study variables. Furthermore, standardized z-scores were used to identify univariate outliers and Mahalanobis distance to detect multivariate outliers. Data screening revealed the absence of both univariate outliers (z score > 3.29) and multivariate outliers (*p* < 0.001 for Mahalanobis distance, Cook’s distance exceeding 1.00) based on the recommended criteria (Tabachnick et al., 2013).

The descriptive statistics and Pearson correlation coefficients are reported in Table 1. Machiavellianism was positively correlated with schadenfreude, moral disengagement and aggressive humor style (*r =* 0.357, 0.223, 0.254, *p <* 0.01), narcissism was positively correlated with schadenfreude, moral disengagement and aggressive humor style (*r =* 0.371, 0.257, 0.274, *p <* 0.01), psychopathy was positively correlated with schadenfreude, moral disengagement and aggressive humor style (*r =* 0.392, 0.298, 0.194, *p <* 0.01), moral disengagement and aggressive humor style were positively correlated with schadenfreude (*r =* 0.642, 0.488, *p <* 0.01).

**Table 1:** Descriptive statistics and correlations among the study variables (*n* = 693).

	Mean	SD	1	2	3	4	5	6
1. Schadenfreude	49.77	23.18	1					
2. Machiavellianism	28.79	7.61	0.357^**^	1				
3. Narcissism	25.71	6.81	0.371^**^	0.432^**^	1			
4. Psychopathy	20.72	5.01	0.392^**^	0.313^**^	0.271^**^	1		
5. Moral disengagement	20.09	8.50	0.642^**^	0.223^**^	0.257^**^	0.298^**^	1	
6. Aggressive humor style	24.07	7.02	0.488^**^	0.254^**^	0.274^**^	0.194^**^	0.419^**^	1

**p < 0.01; SD, Standard Deviation.

### 3.2 Main analysis

**Figure 1:**
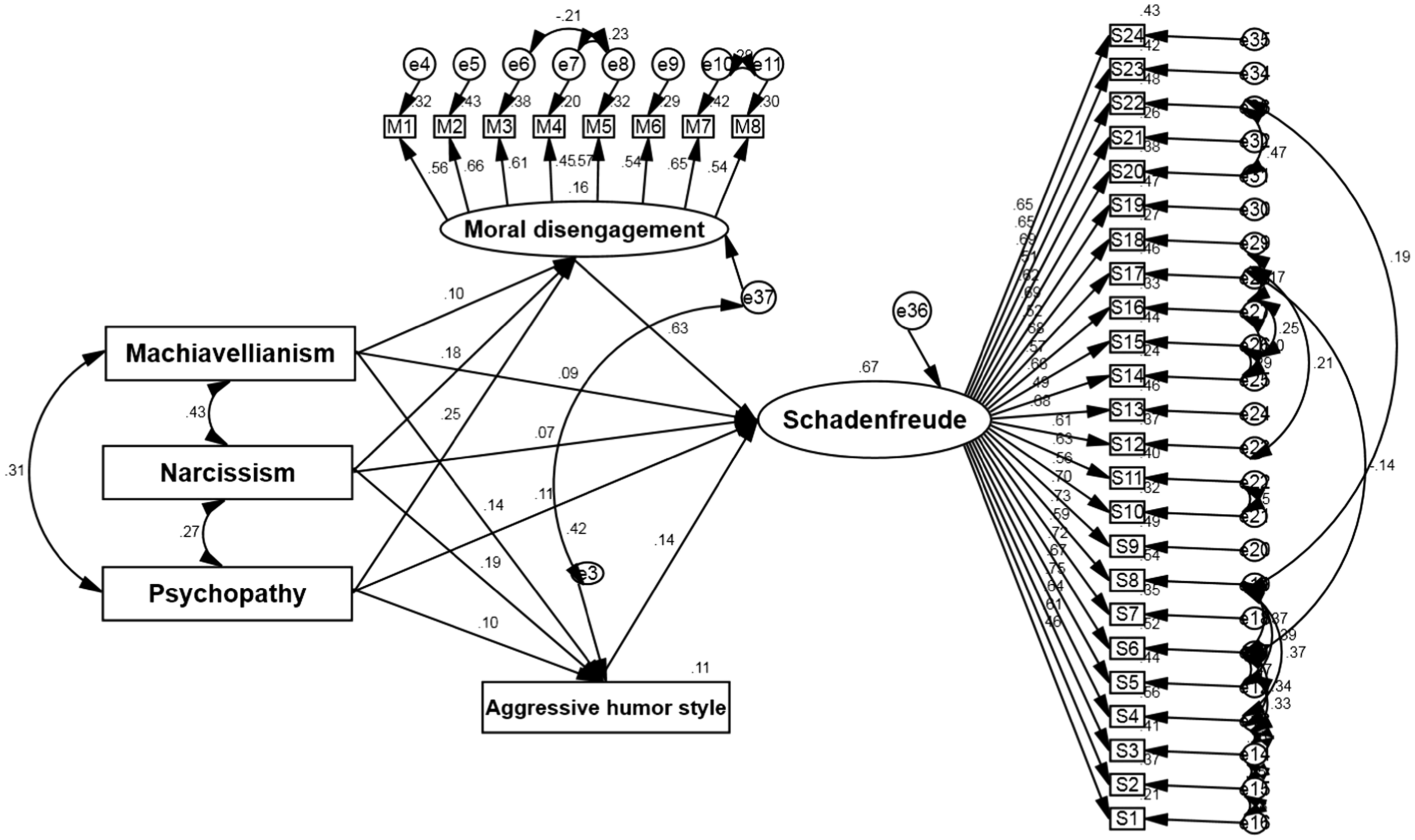
Structural equation model of mediating role of moral disengagement and aggressive humor style in the relationship between Dark Triad traits and schadenfreude; Estimations are standardized betas estimated.

The structural model was calculated and showed sufficient fitness (χ^2^/df = 2.936, CFI = 0.905, TLI = 0.894, IFI = 0.906, RMSEA = 0.053, see Figure 1).

#### 3.2.1 The direct effects

The direct effect of DT traits on schadenfreude was found to be significant (Machiavellianism: β = 0.093, *p* = 0.001, 95% bootstrap confidence interval: 0.040, 0.148, *R*^2^ = 0.124; Narcissism: β = 0.069, *p* = 0.026, 95% bootstrap confidence interval: 0.009, 0.133, *R*^2^ = 0.130; Psychopathy: β = 0.109, *p* = 0.002, 95% bootstrap confidence interval: 0.041, 0.179, *R*^2^ = 0.155). Moral disengagement (β = 0.631, *p* = 0.001, 95% bootstrap confidence interval: 0.522, 0.724, *R*^2^ = 0.649) and aggressive humor style (β = 0.142, *p* < 0.001, 95% bootstrap confidence interval: 0.073, 0.212, *R*^2^ = 0.260) was significantly related to schadenfreude. All direct effects of DT traits on moral disengagement and aggressive humor style were significant. Specifically, the direct effect of narcissism on aggressive humor style (β = 0.186, *p* < 0.001, 95% bootstrap confidence interval: 0.091, 0.277, *R*^2^ = 0.032) as well as the direct effect of psychopathy on moral disengagement (β = 0.252, *p* = 0.001, 95% bootstrap confidence interval: 0.158, 0.331, *R*^2^ = 0.063) were greater than the effects of other DT traits. These findings support hypotheses 1, 2, and 3.

#### 3.2.2 The mediating role of moral disengagement and aggressive humor style

To assess the fourth hypothesis, three indirect effects were examined. The first indirect effect showed that moral disengagement and aggressive humor style significantly mediate the relationship between Machiavellianism and schadenfreude (β = 0.081, *p* = 0.010, 95% bootstrap confidence interval: 0.021, 0.143, *R*^2^ = 0.651). The other indirect effect showed that moral disengagement and aggressive humor style significantly mediate the relationship between narcissism and schadenfreude (β = 0.140, *p* < 0.001, 95% bootstrap confidence interval: 0.073, 0.209, *R*^2^ = 0.645). The third indirect effect showed that moral disengagement and aggressive humor style significantly mediate the relationship between psychopathy and schadenfreude (β = 0.173, *p* < 0.001, 95% bootstrap confidence interval: 0.106, 0.234, *R*^2^ = 0.650). In general, the participants with DT traits were more likely to morally disengage and used an aggressive humor style. Therefore, they were more likely to express schadenfreude. These findings support hypotheses 4.

## 4 Discussion

The present study, consistent with previous research findings (Erzi, 2020; Yee and Lee, 2022), showed that DT traits (Machiavellianism, narcissism, and psychopathy) positively related to schadenfreude, supporting Hypothesis 1. According to appraisal theory, an individual’s assessment of a situation can lead to the expression of schadenfreude when the misfortune of others is perceived as an opportunity for personal gain (Frijda, 2007). Since individuals with DT traits are highly goal-oriented (Jonason and Webster, 2012), their lack of empathy allows them to disregard others’ feelings in pursuit of those goals (Jonason et al., 2013). Therefore, they may perceive another’s misfortune more positively since it helps them advance their own goals.

The findings also revealed a direct effect of the DT traits on moral disengagement and aggressive humor style, supporting Hypothesis 2 and aligning with previous research (Erzi, 2020; Moore et al., 2020; Yee and Lee, 2022) indicating that individuals high in DT traits are more likely to justify their unethical actions and employ aggressive humor styles in their interactions with others. The association between the DT traits and moral disengagement may be attributed to the lack of empathy and callous nature of individuals who exhibit high levels of these traits (Ali et al., 2009; Bader et al., 2021). This results in less compatibility with social norms and a limited capacity for moral reasoning (Connelly et al., 2006), which in turn fosters the development of maladaptive mechanisms such as moral disengagement to justify their behavior.

The observed relationship between the DT traits and aggressive humor style can be attributed to the fact that these constructs share several personality traits, including manipulation, callous, and contempt. Notably, the manipulation of others serves as a common factor between Machiavellianism and aggressive humor style (Vernon et al., 2008). Given that aggressive humor style is characterized by the deliberate belittlement of others through demeaning jokes, individuals with high levels of Machiavellianism may perceive it as a means to manipulate others for personal gain (Martin et al., 2012). Moreover, this humor style, as it is expressed without regard for its potential impact on others, may be more prevalent among individuals with high levels of psychopathy due to their lack of empathy (Veselka et al., 2010). Furthermore, individuals with high levels of narcissism may be more likely to use aggressive humor style, as it allows them to bolster their self-esteem by demeaning others (Zeigler-Hill and Besser, 2011).

In line with Hypothesis 3, the findings of the present study revealed that moral disengagement and aggressive humor style had a direct effect on schadenfreude, which is consistent with the findings of previous research (Erzi, 2020; Yee and Lee, 2022). Schadenfreude, taking pleasure in misfortune, is considered a maladaptive social emotion. While its experience is subjectively positive, expressing joy at others’ misfortunes could be detrimental to social relationships (Rudolph and Tscharaktschiew, 2014). Therefore, it can be argued that moral disengagement likely reduces regret over schadenfreude by justifying immoral behavior and feelings. According to the superiority theory, humor is perceived positively, as it serves as a more acceptable alternative to harsher, unethical expressions of anger such as insults (Hurley et al., 2011). Additionally, the belief others received undeserved rewards can trigger public expressions of resentment toward them (Pietraszkiewicz and Wojciszke, 2014). Based on this, aggressive humor style allows individuals to feel superior by demeaning others (Veselka et al., 2010). Therefore, individuals who frequently adopt this humor style might experience greater pleasure (e.g., laughter) when they perceive their target as humiliated, serving as a temporary boost to their sense of superiority.

Overall, Hypothesis 4, which posited that moral disengagement and aggressive humor style mediate the relationship between DT traits and schadenfreude was supported. Individuals with more DT traits were more likely to morally disengage and used the aggressive humor style. Therefore, they expressed higher levels of schadenfreude. These DT traits might be conceptually-distinct from each other; i.e., Machiavellianism could be explained by a tendency to manipulate others based on organizing self-planning in a way that gains maximum benefit (Lișman and Corneliu, 2023), narcissism by selfishness and self-idealization (Raskin and Terry, 1988) and psychopathy with being emotionally-cold and ignoring the others (Brewer et al., 2015). However, the present results showed that these traits might have similarities because individuals with higher levels of Machiavellianism, narcissism, and psychopathy feel satisfaction from others’ misery by humiliating them. In other words, as the aggressive humor style is used to humiliate and make fun of others without considering its consequences (Martin et al., 2003), enjoying others’ misery using this style of humor for humiliating others increases. Also, these individuals might be morally disengaged, so they can justify their deeds, which are against their moral standards and express schadenfreude more easily. Although schadenfreude is a common emotion, it is considered a conflicting emotion that is avoided, and individuals try to hide its expression (Gromet et al., 2016). Besides, according to Ben-Ze’ev (2014), individuals cannot express schadenfreude when others suffer enormously. In other words, moral disengagement can open a window to justify expressing schadenfreude. Therefore, DT traits make the individual not only ignores other suffering but also enjoy it (Lee and Gibbons, 2017). Furthermore, individuals with elevated DT traits have a tendency to conceal their distinct unpleasantness by using an aggressive style of humor (Martin et al., 2012). As a result, schadenfreude can be seen as a type of social aggression expressed in a humorous manner.

## 5 Conclusions and practical implications

Our study provides evidence that DT traits, comprising Machiavellianism, narcissism, and psychopathy, are positively correlated with schadenfreude, moral disengagement, and aggressive humor style. These findings suggest that individuals with higher levels of DT traits are more likely to derive pleasure from others’ misfortune, justify their immoral behavior, and employ aggressive humor to demean others. Notably, moral disengagement and aggressive humor style were found to mediate the relationship between DT traits and schadenfreude, indicating that individuals with higher levels of DT traits are more likely to morally disengage and use aggressive humor, which in turn increases their experience of schadenfreude. Theoretically, we integrate key constructs from personality, social, and moral psychology literatures, contributing to a more comprehensive framework for understanding the “dark” side of human behavior and emotion, particularly schadenfreude.

Our findings have several practical implications. Firstly, they highlight the importance of understanding the social approaches of individuals with DT traits, which can inform interventions and strategies to mitigate their antisocial behaviors. Secondly, they provide insights into the psychological mechanisms underlying schadenfreude in DT individuals, which can inform clinical assessment and treatment approaches. Additionally, the findings have implications for organizational and social settings, where identifying and addressing DT traits in individuals may be essential for creating a positive and productive work environment. Finally, the results suggest that reducing the prevalence of aggressive humor style on social media may help reduce schadenfreude and promote more positive social interactions.

## 6 Limitations and future directions

Despite the theoretical and practical contributions of our study, there were some limitations requiring consideration when generalizing our findings. The first limitation was associated with the fact that we used a convenient sampling method because we did not have any opportunity to use random selection. This may limit the generalizability of our results. Second, because the study was cross-sectional, it cannot draw any cause-effect inferences. Therefore, future studies should use a longitudinal design to better establish the causal association between DT and schadenfreude. Next, we used a self-report measure, which can generate biases. In future research, we suggest employing objective measures, especially to measure schadenfreude. Furthermore, although the sample size was satisfactory, future studies may employ larger samples with diverse demographic characteristics to enhance the generalizability of the findings. Additionally, different contexts, such as academic and organizational settings, may have distinct motivating factors for schadenfreude, like competition and deservingness, resulting in varying levels of schadenfreude. In order to enrich the literature on schadenfreude, further studies should account for these factors and conduct more precise investigations. Finally, we just looked at the Dark Triad, excluding sadism, which is part of the dark tetrad. Incorporating the dark tetrad, as well as gluckschmerz (suffering from others’ good fortune), could broaden the theoretical model and provide a more complete picture of the dark side of human behavior and emotion.

## Data availability statement

The raw data supporting the conclusions of this article will be made available by the authors, without undue reservation.

## Ethics statement

The studies involving humans were approved by the Research Ethics Committee of the Faculty of Psychology and Education, Tehran University (Ethics ID: IR.UT.PSYEDU.REC.1401.043). The studies were conducted in accordance with the local legislation and institutional requirements. The participants provided their written informed consent to participate in this study.

## Author contributions

MS: Conceptualization, Data curation, Formal analysis, Investigation, Methodology, Software, Writing – original draft. RP: Conceptualization, Project administration, Supervision, Writing – review & editing. ZA: Conceptualization, Project administration, Supervision, Writing – review & editing.
